# Perceptions and satisfaction among couples on male involvement during pregnancy: a cross-sectional study

**DOI:** 10.3389/fpubh.2025.1625849

**Published:** 2025-10-22

**Authors:** Chuanya Huang, Dongning He, Yirong He, Yuan Li, Yayi Hu, Guoyu Wang, Biru Luo, Jianhua Ren

**Affiliations:** ^1^Department of Gynecology and Obstetrics Nursing, West China Second University Hospital, Sichuan University, Chengdu, Sichuan, China; ^2^Key Laboratory of Birth Defects and Related Diseases of Women and Children, Sichuan University, Ministry of Education, Chengdu, Sichuan, China; ^3^Department of Nursing, West China Second University Hospital, Sichuan University, Chengdu, Sichuan, China; ^4^Department of Gynecology and Obstetrics, West China Second University Hospital, Sichuan University, Chengdu, China

**Keywords:** male involvement, maternal health, satisfaction, perception, pregnancy

## Abstract

**Introduction:**

Previous studies showed that male involvement during pregnancy could improve maternal and newborn outcomes. However, little is known about the perceptions and satisfaction of both men and women regarding male involvement during pregnancy. Comprehending various viewpoints could help to design tailored interventions for better male involvement. This study aims to survey the perception and satisfaction of both men and women with male involvement during pregnancy in China.

**Methods:**

This cross-sectional study was carried out in Chengdu, China. A total of 1,357 couples were surveyed using a self-administered questionnaire that assessed both men’s and women’s perception of male involvement in pregnancy-related activities, as well as their respective satisfaction with the male involvement in these activities. Descriptive statistics, paired t-tests, McNemar tests, and multiple stepwise regression analyses were employed to analyze the data.

**Results:**

Men reported higher involvement in pregnancy-related activities (9.67 ± 1.46) than women perceived (9.00 ± 1.74), with significant differences across all activities (*p* < 0.05). Similarly, men reported greater satisfaction with their involvement (46.80 ± 8.47) than women (43.90 ± 8.86). Regression analysis showed that the gravidity, ethnic group of men and women, the number of miscarriages, the place of residence of men, and education level of men were associated with men’s perception of male involvement during pregnancy. In addition, the age of men, the place of residence of women, and ethnic group of men and women were found to be factors associated with women’s perception of male involvement during pregnancy.

**Conclusion:**

Men reported higher involvement and satisfaction than women perceived, revealing a perceptual gap. To address this, healthcare programs should encourage joint antenatal counseling, foster couple-centered communication, and provide community education, while training providers to facilitate constructive dialogue between men and women.

## 1 Introduction

Pregnancy is an essential stage for maternal health and fetal development, as exposures and health-related behaviors during this period may exert long-lasting effects on both women and children. The World Health Organization (WHO) advocated that pregnancy should be a positive experience, ensuring women and their babies reach their full potential for health and well-being (1). Women undergo significant physical and mental changes during pregnancy (2–4). These changes may result in a variety of physiological and psychological stressors. Therefore, emotional and physical support from men is crucial to help women cope effectively during pregnancy. Male involvement during pregnancy is defined as men’s active engagement in emotional support, financial provision, household responsibilities, shared decision-making, and participation in antenatal care (5). Research has demonstrated that male involvement during pregnancy benefits maternal health by reducing physical burdens, enhancing nutrition, improving antenatal care attendance, and decreasing anxiety and depressive symptoms (6–8). Given these benefits, the WHO recommended active male involvement during pregnancy as one of the effective strategies for improving maternal and newborn health (9).

Despite these recommendations, male involvement during pregnancy remains limited in many low- and middle-income countries. For instance, Falade-Fatila et al. reported that 56.9% of men had good involvement in pregnancy-related care in Nigeria (10), and a study by Elizabeth et al. showed that 56.9% of women attended antenatal care with their husbands in Mbeya (11). Recent findings from Ethiopia indicate that men’s educational level, couples’ living status, attitude towards antenatal care, and gender norms may significantly influence their involvement (12, 13). Similarly, Saumya et al. reported that in Tanzania, male involvement during pregnancy was constrained by gender norms and structural barriers, including work obligations and prolonged clinic wait times (14). Collectively, these studies underscore how social and cultural contexts critically shape male involvement in pregnancy across low- and middle-income settings. However, to the best of our knowledge, no study has been conducted in China to examine this issue, which makes it difficult to develop targeted interventions to improve male involvement in this context. Limited evidence is available from a cross-sectional study in Singapore, where approximately 70% of the population is Chinese; this study reported that only 35.2% of men demonstrated a high level of involvement during pregnancy (15). Given the similar cultural background, these findings raise concerns that male involvement in China may also be suboptimal, highlighting the urgent need for context-specific research. In addition, previous studies often investigated male involvement during pregnancy only from men or women, but the single-gender perspective might have limitations. A study conducted in Japan that focused on the feelings of first-time couples and the satisfaction of women with men’s support for their pregnancy found that some men were worried about the physical burden on their wives during pregnancy, but women did not perceive that men were worried about these things (16), which suggests that there may be differences in how men and women perceive the same thing. This highlights the need for a dyadic approach that captures both men’s and women’s perceptions simultaneously, in order to provide a more accurate and comprehensive understanding of male involvement.

Maryam et al.’s study demonstrated that maternal stress levels and gestational age were significantly associated with women’s satisfaction with the emotional and financial support received from their husbands during pregnancy (17, 18). However, there is still a lack of research on couples’ satisfaction with male involvement during pregnancy, and it remains unclear which pregnancy-related activities couples are more satisfied with regarding male participation, and which activities have lower satisfaction. Investigating the perceptions and satisfaction of both men and women regarding male involvement during pregnancy might assist researchers in designing targeted interventions to promote better male involvement during pregnancy. Therefore, the primary objective of this study was to explore men’s and women’s perceptions and satisfaction regarding male involvement during pregnancy and to analyze whether there were differences between their perceptions and satisfaction, particularly in specific pregnancy-related activities. Additionally, this study sought to investigate the current status of male involvement during pregnancy in China, a topic that had been underexplored in previous research. Finally, this study explored factors that may influence couples’ perception of male involvement. These findings have practical implications. Understanding couples’ perceptions and satisfaction regarding male involvement could help healthcare providers, policymakers, and community programs develop targeted strategies to support active male involvement, enhance maternal well-being, and improve pregnancy outcomes. Moreover, identifying activities with lower satisfaction can inform interventions that address specific gaps in support, ultimately promoting more effective engagement of men during pregnancy.

## 2 Methods

### 2.1 Study design and settings

This study was a cross-sectional study conducted in five maternity wards of a women’s and children’s hospital in Chengdu City, Sichuan Province of China from January 2022 to August 2022. This hospital is the largest women’s and children’s hospital in Sichuan Province and attracts patients from different cities in Southwest China, with approximately 25,000 births per year. Hence, this hospital could provide a population with various sociodemographic backgrounds for this study.

### 2.2 Sampling and participant

The sampling method used in this study was convenience sampling. Participants were women who had recently given birth (between 1 day and 1 month postpartum) in the five maternity wards of the hospital and their spouses. Convenience sampling was chosen because postpartum women and their husbands have a limited hospitalization period and are relatively difficult to recruit, and approaching them directly in the wards ensured timely enrollment and adequate participation. We first screened women who met the inclusion and exclusion criteria in the hospital’s information system. Then the researchers (HCY, HDN, and HYR) introduced the purpose of the study in the wards and invited them and their spouses to participate in the study. Those who were willing to participate in this study would sign an informed consent form.

The inclusion criteria of participants were: (1) women’s age ≥ 20 years, men’s age ≥ 22 years, (2) gestational age of women ≥ 37 weeks, and (3) between 1 day and 1 month after birth. For ethical considerations, the exclusion criteria of participants were: (1) this birth was a stillbirth, (2) their newborn had died within the 24 h after birth, (3) women had severe complications during pregnancy and/or after birth, or (4) they had a history of severe mental disorders.

The sample size calculation formula for cross-sectional studies was used to calculate the sample size of this study (19). The sample size for this study was calculated based on the proportion of high levels (53.9%) of male involvement during pregnancy reported in the literature (20), with *α* = 0.05, and *δ* = 0.03. Hence, the calculated sample size was 1,092. Taking into account the sampling error, the sample size was increased by 10% to 1,200. Since this study surveyed both women and their spouses, the minimum sample size required was 1,200 couples.

### 2.3 Instruments

#### 2.3.1 Basic information of participants

Sociodemographic data of couples and clinical data related to pregnancy and birth of women were collected. Sociodemographic data include age, ethnic group, place of residence, education level, occupation, individual monthly income, length of marriage, and persons living together during pregnancy. Clinical data include height, last prenatal weight, mode of birth, previous pregnancy, parity, history of miscarriage, pregnancy complications, and whether *in vitro* fertilization-embryo transfer (IVF-ET) was used.

#### 2.3.2 Questionnaire on male involvement during pregnancy

A self-administered questionnaire was developed to collect data on male involvement during pregnancy. The development of the questionnaire was conceptually guided by the systematic review of Galle et al. (5), which synthesized the domains and activities of male involvement in maternal health, from which we extracted relevant indicators in the literature to design the primary questionnaire. To test the content validity index, we invited 7 experts, consisting of 3 obstetricians, 2 midwives, and 2 nurses, to give their expert opinions on the primary questionnaire. Then, we revised the questionnaires according to the advice of experts. After 2 weeks, we invited the same 7 experts to review the questionnaire. In addition, we selected 20 couples in the maternity wards who were not involved in the subsequent data analysis to test the reliability of the questionnaire and ensure that the content of the questionnaire could be understood by the participants. The questionnaire can be found in the Supplementary File 1.

The questionnaire consisted of 22 questions related to the 11 activities that men can be involved in during pregnancy, as well as their satisfaction with participating in each activity. These activities, including attending antenatal care visits, providing financial support, offering emotional support, and assisting with household tasks, among others, represent forms of male involvement during pregnancy. If the male participated in an activity, it was scored as 1 point; otherwise, 0 point. Finally, the scores of each item were added together to get the total score, ranging from 0 to 11 points. The higher the total score, the better the male involvement during pregnancy. In addition, the women and their spouses were required to evaluate their satisfaction with male involvement in each activity using the five-point Likert scale: very satisfied = 5 points, satisfied = 4 points, average = 3 points, dissatisfied = 2 points, and very dissatisfied = 1 point. The scores of each item were added together to get the total satisfaction scores, ranging from 5 to 55 points. Also, the higher the total score, the higher the satisfaction with the male involvement during pregnancy.

Participants could complete this questionnaire in 5 to 15 min. The item-content validity index of the questionnaire was between 0.857 and 1.000, the scale-content validity index was 0.974, and the Cronbach’s alpha was 0.823.

### 2.4 Data collection

Data were collected using the Wenjuanxing platform (an online questionnaire platform that could generate links and QR codes of questionnaires). To avoid missing data, each question was set as mandatory. Before the formal survey, the investigators were trained to guarantee that they could explain the purpose and answer the related questions from participants of this study. The participants who were willing to participate in this study would use their phones to scan the QR codes of the questionnaire. To minimize discussions between couples that could affect responses, the investigators remained nearby in the ward to provide guidance if needed until both participants submitted the questionnaire successfully. Each patient in the hospital had a unique registration number, which was collected together with the questionnaire so that couples could be matched by the registration number, and the registration number was also used to collect clinical data in the hospital information management system by researchers.

### 2.5 Data analysis

SPSS 27.0 software was used to analyze the data, and all tests were considered statistically significant when two-sided *p* < 0.05. First, the distribution of continuous data was tested. Then, data that conformed to normal distribution were described using mean ± standard deviation (Mean ± SD); data that did not conform to normal distribution were described using median (P25, P75). In addition, categorical data were described as numbers (*n*) and percentages (%). The McNemar test and paired *t*-test were used to analyze paired nominal data and continuous data, respectively. To investigate potential sociodemographic and clinical factors associated with men’s and women’s perception of male involvement during pregnancy, we applied multiple linear stepwise regression. This method was selected because our study was exploratory and involved a relatively large set of candidate variables; stepwise regression allowed us to identify the most relevant predictors in a parsimonious way.

### 2.6 Ethics approval

This study was approved by the Ethics Committee of West China Second University Hospital, Sichuan University, and the ethics approval number is 2021 (168). Informed consent was obtained from all participants, and they could withdraw from the study at any time, but their treatment or care would not be affected in any way.

## 3 Results

### 3.1 Sociodemographic information of participants

Between January 2022 and August 2022, we recruited 1,357 couples to participate in this study. The sociodemographic information of participants is shown in Table 1. The age of men ranged from 24 to 47 years, with an average of 32.54 ± 3.76 years; the age of women ranged from 23 to 45 years, with an average of 31.20 ± 3.55 years. The Han ethnic group accounted for 95.6% of men and 97.3% of women. The proportion of women with postgraduate education (20.9%) was slightly higher than that of men (19.5%). In the group with a monthly income of more than ¥10,000, the proportion of men (51.2%) was much higher than that of women (22.3%). The proportion of both women and men engaged in professional/teachers and sales/service was the largest (39.1 and 36.8%, respectively), but the proportion of unemployed women was 14.7%. 66.0% of couples lived with their spouses during pregnancy. The median length of marriage was 3 years, ranging from 0 to 18 years.

**Table 1:** Sociodemographic information of couples (*N* = 2,714).

Variables	Men (*n* = 1,357)	Women (*n* = 1,357)
Age (years)^a^, Mean ± SD	32.54 ± 3.76	31.20 ± 3.55
Ethnic group		
Han	1,297 (95.6)	1,321 (97.3)
Others	60 (4.4)	36 (2.7)
Place of residence		
Urban	1,288 (94.9)	1,300 (95.8)
Rural	69 (5.1)	57 (4.2)
Education level		
Postgraduate	264 (19.5)	283 (20.9)
Undergraduate	702 (51.7)	707 (52.1)
College	273 (20.1)	253 (18.6)
High school	82 (6.0)	90 (6.7)
Secondary School and below	36 (2.7)	24 (1.8)
Individual monthly income, ¥		
>10,000	696 (51.2)	303 (22.3)
5,000–10,000	476 (35.0)	531 (39.1)
3,000–5,000	151 (11.1)	314 (23.1)
1,000–3,000	29 (2.1)	73 (5.4)
<1,000	8 (0.6)	136 (10.0)
Occupation		
Professional/Teachers	531 (39.1)	499 (36.8)
Managerial/Executive	280 (20.6)	236 (17.4)
Sales/Service	359 (26.5)	299 (22.1)
Workers	25 (1.8)	17 (1.3)
Farmers	114 (8.4)	86 (6.4)
Students	8 (0.6)	8 (0.6)
Not working	28 (2.1)	200 (14.7)
Others	12 (0.9)	12 (0.9)
Persons living together during pregnancy		
Alone	29 (2.1)	23 (1.7)
With spouse	896 (66.0)	896 (66.0)
With spouse and other family members	405 (29.8)	408 (30.1)
With other family members only	27 (2.0)	30 (2.2)
Length of marriage (years)^b^, Median (P25, P75)	3.0 (2.0, 6.0)	

SD, standard deviation; ¥, Chinese Yuan.

^a^Indicates data with a normal distribution, presented as Mean ± SD; ^b^Indicates data with a non-normal distribution, presented as Median (P25, P75).

### 3.2 Clinical characteristic of women

The clinical characteristic of women is shown in Table 2. The median height of women was 1.60 m, the median prenatal last weight was 67.00 Kg, and the median prenatal last BMI was 26.30 Kg/m^2^. In terms of the mode of birth, 67.1% of the women had cesarean section and 32.9% had natural birth. Nearly half of the women had zero previous pregnancies (52.7%), and 78.2% of the women had zero previous births. In terms of pregnancy-related comorbidities and complications, 1,007 women (74.2%) had pregnancy complications and 376 women (27.7%) had pregnancy complications. The proportion of couples who used IVF-ET was 14.1%.

**Table 2:** Clinical characteristic of women (*n* = 1,357).

Variables	*n* (%)	Median (P25, P75)	Range
Height (m)		1.60 (1.56, 1.63)	1.43 ~ 1.75
Prenatal last weight (Kg)		67.00 (62.00, 72.50)	48 ~ 103
Prenatal last BMI (Kg/m^2^)		26.30 (24.56, 28.20)	19.81 ~ 34.82
Mode of birth			
Cesarean section	910 (67.1)		
Natural birth	447 (32.9)		
Gravidity			
0	715 (52.7)		
1	310 (22.8)		
2 and above	332 (24.5)		
Parity			
0	1,061 (78.2)		
1	276 (20.3)		
2 and above	20 (1.5)		
Number of miscarriages			
0	831 (61.2)		
1	302 (22.3)		
2 and above	224 (16.5)		
Complications during pregnancy			
Yes	1,007 (74.2)		
No	350 (25.8)		
Complications of pregnancy			
Yes	376 (27.7)		
No	981 (72.3)		
IVF-ET			
Yes	192 (14.1)		
No	1,165 (85.9)		

m, meter; Kg, kilogram; BMI, body mass index; IVF-ET, *in vitro* fertilization & embryo transfer.

### 3.3 Perception and satisfaction of male involvement during pregnancy

The McNemar test was used to analyze the differences in the couples’ perception of whether men were involved in each activity. In the 11 activities, the proportion of men who self-evaluated their involvement in these activities was higher than that of women, and the difference was statistically significant (*p* < 0.05). This study also used a paired *t*-test to analyze the difference between the men’s self-evaluation of participating in activities and how they were perceived by women. The results showed that the total score of involvement evaluated by men was 9.67 ± 1.46, and perceived by women was 9.00 ± 1.74, with a statistically significant difference (*p* < 0.001).

In terms of satisfaction with male involvement during pregnancy, men scored higher than women in satisfaction scores for all 11 activities as well as in the total score, and the differences were statistically significant (*p* < 0.05). Both men and women (4.53 ± 0.78 and 4.33 ± 0.90) reported the highest satisfaction with male involvement in accompanying antenatal care, while their satisfaction (3.71 ± 1.37 and 3.32 ± 1.27) was the lowest with attending antenatal health education together (Table 3).

**Table 3:** Couples’ perception and satisfaction of male involvement during pregnancy (*N* = 2,714).

Items	Couples’ perceptions of male involvement during pregnancy			Couples’ satisfaction of male involvement during pregnancy		
	Men (*n* = 1,357)	Women (*n* = 1,357)	*P*	Men (*n* = 1,357)	Women (*n* = 1,357)	*P*
Accompanying antenatal care						
	1,310 (96.5)	1,286 (94.8)	<0.001^a^	4.53 ± 0.78	4.33 ± 0.90	<0.001
Discussing antenatal care results with women						
	1,341 (98.8)	1,286 (94.8)	<0.001^a^	4.39 ± 0.93	3.98 ± 1.06	<0.001
Consulting medical personnel about antenatal care						
	1,046 (77.1)	755 (55.6)	<0.001^a^	3.90 ± 1.28	3.47 ± 1.26	<0.001
Financial support						
	1,307 (96.3)	1,250 (92.1)	<0.001^a^	4.38 ± 0.93	4.13 ± 0.96	<0.001
Transportation support						
	1,261 (92.9)	1,249 (92.0)	<0.001^a^	4.45 ± 0.92	4.16 ± 1.01	0.018
Nutritional support						
	1,242 (91.5)	1,225 (90.3)	<0.001^a^	4.32 ± 1.05	4.23 ± 1.01	<0.001
Emotional support						
	1,318 (97.1)	1,301 (95.9)	<0.001^a^	4.41 ± 0.92	4.27 ± 0.95	<0.001
Household support						
	1,281 (94.4)	1,221 (90.0)	<0.001^a^	4.32 ± 1.03	4.18 ± 1.04	<0.001
Participating in the decision-making for the mode of birth						
	1,133 (83.5)	1,097 (80.8)	<0.001^a^	4.23 ± 1.13	4.03 ± 1.12	<0.001
Attending antenatal health education together						
	713 (52.5)	547 (40.3)	0.005^a^	3.71 ± 1.37	3.32 ± 1.27	<0.001
Actively learning about pregnancy-related knowledge						
	1,190 (87.7)	978 (72.1)	<0.001^a^	4.17 ± 1.13	3.78 ± 1.23	<0.001
Total scores						
	9.67 ± 1.46	9.00 ± 1.74	<0.001^b^	46.80 ± 8.47	43.90 ± 8.86	<0.001

^a^Denotes that the McNemar test was used, and ^b^Denotes that the paired t-test was used.

### 3.4 Regression analyses examining covariates of perception of male involvement during pregnancy

Table 4 shows the result of multiple linear stepwise regression analyses. Model 1 is the multiple linear stepwise regression model for men’s perception of male involvement during pregnancy, and Model 2 is for women’s perception of male involvement during pregnancy. According to the results of multiple linear stepwise regression, the gravidity (*β* = −0.210, *p* < 0.001), ethnic group of women (*β* = −0.098, *p* < 0.001), and ethnic group of men (*β* = 0.089, *p* = 0.001) significantly influenced men’s perception of male involvement during pregnancy. Additionally, the number of miscarriages (*β* = 0.132, *p* = 0.022), place of residence of men (*β* = −0.076, *p* = 0.008), and education level of men (*β* = −0.069, *p* = 0.015) were also associated with men’s perception of male involvement during pregnancy. In the analysis of women’s perception, the age of men (*β* = −0.091, *p* < 0.001), place of residence of women (*β* = −0.068, *p* = 0.011), ethnic group of men (*β* = 0.083, *p* = 0.003), and ethnic group of women (*β* = −0.080, *p* = 0.004) were found to be factors associated with perception of male involvement during pregnancy.

**Table 4:** Regression analyses examining covariates of perception of male involvement during pregnancy.

Variables	Value	B	SE	*β*	*t*	*P*
Model 1						
(Constant)		10.731	0.306	–	35.110	<0.001
Gravidity		−0.369	0.101	−0.210	−3.651	<0.001
Ethnic group (women)	Han (Ref.)	−0.885	0.251	−0.098	−3.523	<0.001
	Others					
Ethnic group (men)	Han (Ref.)	−0.631	0.196	0.089	3.222	0.001
	Others					
Number of miscarriages		0.253	0.111	0.132	2.287	0.022
Place of residence (men)	Urban (Ref.)	−0.504	0.188	−0.076	−2.673	0.008
	Rural					
Education level (men)	≤ Secondary School (Ref.)	0.110	0.045	−0.069	−2.430	0.015
	> Secondary School					
Model 2						
(Constant)		10.982	0.481	–	22.836	<0.001
Age, years (men)		−0.042	0.012	−0.091	−3.367	<0.001
Place of residence (women)	Urban (Ref.)	0.593	0.234	−0.068	−2.533	0.011
	Rural					
Ethnic group (men)	Han (Ref.)	−0.704	0.235	0.083	3.000	0.003
	Others					
Ethnic group (women)	Han (Ref.)	−0.865	0.300	−0.080	−2.881	0.004
	Others					

Model 1: F = 7.834, *P* < 0.001; Durbin-Watson = 1.919; *R*^2^ = 0.204, adjusted *R*^2^ = 0.198. Model 2: F = 8.088, *P* < 0.001; Durbin-Watson = 1.976; *R*^2^ = 0.265, adjusted *R*^2^ = 0.254.

## 4 Discussion

This study investigated the perceptions and satisfaction of male involvement during pregnancy from the perspectives of both men and women and explored the associated sociodemographic and clinical factors. Several important findings emerged. First, men consistently rated their involvement and satisfaction higher than women’s perceptions of their men’s participation. Second, the highest satisfaction was reported for men accompanying women to antenatal care visits, whereas the lowest satisfaction was for attending antenatal health education together. Third, both men’s and women’s perceptions of male involvement were influenced by multiple factors, including ethnicity, place of residence, education level, gravidity, and reproductive history.

### 4.1 Current status of male involvement

From both the men’s and women’s perspectives, the overall level of male involvement during pregnancy was relatively high, as reflected by the total scores of reported involvements. However, participation differed across specific pregnancy-related activities. For example, regarding one of the most commonly used indicators in previous studies, which is whether men accompany their wives to antenatal care visits (5, 21, 22), our results showed remarkably high participation rates. Our results showed that 96.5% of men and 94.8% of women reported that men involved in accompanying antenatal visits, respectively. These proportions were higher than the previous report. For instance, a cross-sectional study conducted by a multi-agency regional joint program on gender-based violence prevention in the Asia-Pacific region, revealed that 87.2% of Chinese men, 88.7% of Indonesian men, 54.5% of Bangladeshi men, and 56.1% of men in Sri Lanka had accompanied their wives for antenatal care (23). Paul et al.’s study showed that 85% of men had accompanied their wives to antenatal care contacts in India (24). These differences may stem from variations in health system organization, the degree of policy prioritization of maternal health, and culturally embedded gender norms that shape men’s perceived roles during pregnancy (25). In this study, apart from accompanying antenatal care, men also showed higher involvement rates in other pregnancy-related activities, such as financial support and emotional support. This result may also be related to the marital culture in the study settings. The core of this cultural norm involves showing more respect and care for women within the family and taking on more household responsibilities. The cross-country contrast highlights that “accompanying antenatal care visits” may serve as a sensitive indicator of paternal engagement, reflecting not only individual willingness but also structural and cultural facilitators (26).

However, male involvement in some activities, such as consulting medical personnel about antenatal care and attending antenatal health education together, was lower compared to other activities. This may be attributed to healthcare institutions often excluding men from antenatal care services. Studies in developing countries showed that many hospitals did not allow men to accompany their wives for antenatal care and related health education (27–29), which partly results in men being unable to participate in these activities. In Ghana, men also reported that while they may accompany their wives, there are no services specifically targeting men, and they are largely passive (just waiting) rather than being involved (30). Chiang et al., in a qualitative systematic review, reported that while antenatal care was frequently perceived by men as a domain reserved for women, a proportion of them nonetheless showed an intention to engage in such services in low- and middle-income settings (31). Therefore, antenatal healthcare programs should emphasize the involvement of men and include them in activities such as antenatal care and health education, thereby improving their involvement in these activities.

### 4.2 Discrepancies between men’s and women’s perceptions and satisfaction of involvement

This study assessed how both men and women perceive and feel satisfied with male involvement in pregnancy-related activities. Our findings indicated that men consistently rated their own involvement more positively than women perceived it, with statistically significant differences observed across all types of pregnancy-related activities. In other words, men considered themselves to be more supportive than women reported them to be. These results suggest that the support provided by men during pregnancy may not always be recognized or perceived by their wives. Several factors may account for this discrepancy. First, since participants were surveyed postpartum, recall bias may distort memory of specific acts or frequency. Second, men and women may have different interpretations of what “involvement” entails: men may consider logistical or financial support sufficient, while women may expect emotional, informational, or physical assistance (32, 33). Third, communication gaps between spouses about expectations and roles may lead to a mismatch in perception and satisfaction (34).

These perception gaps may not be unique to our setting. For example, in Uganda, men described the health care system as “unwelcoming” and reported feeling excluded even when attempting to participate, which likely reduces how their involvement is perceived by both themselves and their partners (35). In Bangladesh urban slums, many women reported that even when their husbands accompany them or help, discussions with health workers and visible participation vary, influencing women’s satisfaction with support (36). Similarly, studies in Tanzania indicate that traditional gender norms affect what roles men take, which in turn affects how both spouses perceive these roles (37). The presence of these discrepancies matters because women’s perceptions of inadequate support, even if men believe they are doing enough, can reduce women’s satisfaction, emotional well-being, and possibly impact utilization of prenatal services. To address this, future interventions should explicitly measure both men’s and women’s perceptions, encourage open communication about roles, standardize definitions of “involvement” in research, and train health providers to facilitate male involvement in ways that are visible, meaningful, and acknowledged by both spouses.

### 4.3 Sociodemographic and clinical correlates of perceived involvement

The findings indicate that men residing in urban areas perceived greater male involvement during pregnancy compared to those in rural areas. These results are consistent with the study by Rumaseu et al. (38), which similarly highlighted this urban–rural divide. One plausible explanation is that men in rural areas often face relatively limited access to healthcare services, resources, and information, which may reduce their capacity to support their wives during pregnancy. Furthermore, Lowe’s study identified gender perception as a significant barrier to male participation in pregnancy-related activities in rural areas. Specifically, many men in these regions view pregnancy and childbirth as within the traditional domain of women and consider domestic responsibilities during pregnancy as not warranting their efforts (39). In contrast, Sodeinde et al. (40) conducted a cross-sectional study in Nigeria, comparing male involvement in childbirth preparation between rural and urban areas. Their findings revealed statistically significantly higher male involvement in rural areas than in urban areas. However, they also noted that higher levels of education were a key predictor of male involvement in childbirth preparation across both settings. This suggests that, regardless of geographic location, men with higher educational attainment tend to be more engaged in childbirth preparation.

The study also identified the ethnic group as a factor influencing male involvement during pregnancy. Specifically, women in the Han ethnic group reported receiving more support from their husbands compared to women from other ethnic groups. Similarly, Nyasiro et al. (20) found that men from the Gogo ethnic group in central Tanzania were 1.495 times more likely to participate in antenatal care services compared to men from other ethnic groups. Thus, the ethnic group appears to exert a notable influence on male involvement during pregnancy, and whether this influence is positive or negative may depend on gender norms embedded within ethnic cultures, as well as attitudes towards pregnancy and childbirth (41). However, due to the limited representation of other ethnic groups in this study, specific subgroup analyses were not conducted. Future research should further explore male involvement across different ethnic groups and examine the factors that shape this involvement.

Additionally, the results demonstrated that husbands of multiparous women were less engaged in pregnancy-related activities than those of primiparous women. Similar findings were reported in Kakaire et al.’s study (42), suggesting that men may provide more selective support during subsequent pregnancies, possibly due to increased confidence and familiarity gained from prior pregnancy and childbirth experiences. Moreover, this study found that male involvement was higher among couples with a history of miscarriage. This may suggest that previous pregnancy loss prompts men to place greater emphasis on subsequent pregnancies, resulting in more active and supportive involvement during pregnancy-related activities.

### 4.4 Strengths and limitations

This is the first study that surveyed both men’s and women’s perceptions and satisfaction of male involvement during pregnancy in China, which not only showed the current status of male involvement during pregnancy in China but also demonstrated the differences in perception and satisfaction between men and women. Based on the results of this study, we proposed that the research in this field should not be focused on only men or women, but on both sides. However, this study also has some limitations. First, since the survey was conducted postpartum, it is difficult to avoid some recall bias. Second, as this study employed convenience sampling and was conducted at a single hospital, the generalizability of the findings may be limited. Third, due to the lack of widely used questionnaires for evaluating male involvement during pregnancy in previous research, a self-designed questionnaire was used in this study, which may result in an incomplete assessment of male involvement during pregnancy. Therefore, this study suggests that future research could design questionnaires for male involvement during pregnancy that could be widely used and conduct multicenter surveys. Furthermore, we acknowledge that stepwise regression has limitations, such as the risk of overfitting, reliance on the specific dataset, and the possibility of excluding variables that may be theoretically important. Therefore, the identified predictors should be interpreted with caution and further validated in future studies with larger and more diverse samples.

## 5 Conclusion

The results showed that men reported higher perception and satisfaction with male involvement during pregnancy than women, highlighting a perceptual gap between couples. To address this gap, healthcare settings should implement strategies such as joint antenatal counseling to increase maternal awareness of men’s roles, promote couple-centered communication in pregnancy-related decision-making, provide community education programs emphasizing the importance of male involvement, and train healthcare providers to facilitate constructive dialogue between men and women. These strategies can enhance male participation, improve maternal satisfaction, and ultimately support better maternal and newborn outcomes.

## Data Availability

The raw data supporting the conclusions of this article will be made available by the authors, without undue reservation.

## References

[1] World Health Organization. Maternal health. (2024). Available online at: https://www.who.int/, https://www.who.int/health-topics/maternal-health#tab=tab_1 (accessed June 11, 2024).

[2] Mackenzie J Murray E Lusher J. Women's experiences of pregnancy related pelvic girdle pain: a systematic review. Midwifery. (2018) 56:102–11. doi: 10.1016/j.midw.2017.10.011, PMID: 29096278

[3] van de Loo KF Vlenterie R Nikkels SJ Merkus PJ Roukema J Verhaak CM. Depression and anxiety during pregnancy: the influence of maternal characteristics. Birth. (2018) 45:478–89. doi: 10.1111/birt.12343, PMID: 29517137

[4] Wu H Sun W Chen H Wu Y Ding W Liang S. Health-related quality of life in different trimesters during pregnancy. Health Qual Life Outcomes. (2021) 19:1–11. doi: 10.1186/s12955-021-01811-y 34289867 PMC8296584

[5] Galle A Plaieser G Van Steenstraeten T Griffin S Osman NB Roelens K. Systematic review of the concept 'male involvement in maternal health' by natural language processing and descriptive analysis. BMJ Glob Health. (2021) 6:e004909. doi: 10.1136/bmjgh-2020-004909, PMID: 33846143 PMC8048011

[6] Tokhi M Comrie-Thomson L Davis J Portela A Chersich M Luchters S. Involving men to improve maternal and newborn health: a systematic review of the effectiveness of interventions. PLoS One. (2018) 13:e0191620. doi: 10.1371/journal.pone.0191620, PMID: 29370258 PMC5784936

[7] Faramarzi M Shariatpanahi M Mahboubeh Mirtabar S Barat S Nasiri Amiri F Khoozan M. Expectations and participatory performance of husbands in improvement of anxiety disorders in pregnant women: a qualitative study. Perspect Psychiatr Care. (2023) 2023:8991842. doi: 10.1155/2023/8991842

[8] Sanaati F Mohammad-Alizadeh Charandabi S Farrokh Eslamlo H Mirghafourvand M Alizadeh Sharajabad F. The effect of lifestyle-based education to women and their husbands on the anxiety and depression during pregnancy: a randomized controlled trial. J Matern Fetal Neonatal Med. (2017) 30:870–6. doi: 10.1080/14767058.2016.1190821, PMID: 27186630

[9] World Health Organization. WHO recommendations on health promotion interventions for maternal and newborn health 2015. Switzerland: World Health Organization (2015). 26180864

[10] Falade-Fatila O Adebayo AM. Male partners' involvement in pregnancy related care among married men in Ibadan, Nigeria. Reprod Health. (2020) 17:1–12. doi: 10.1186/s12978-020-0850-2 31992315 PMC6988334

[11] Kabanga E Chibwae A Basinda N Morona D. Prevalence of male partners involvement in antenatal care visits - in Kyela district, Mbeya. BMC Pregnancy Childbirth. (2019) 19:1–6. doi: 10.1186/s12884-019-2475-4 31477058 PMC6720074

[12] Gessesse NA Gela GB Aweke AM Beyene FY Kassahun EA Getu AA. Male partners’ involvement in antenatal care and its associated factors in west-Central Ethiopia. BMC Public Health. (2024) 24:3015. doi: 10.1186/s12889-024-20502-z, PMID: 39482659 PMC11526563

[13] Muhabaw T Hailemeskel S Lambebo A. Male involvement in antenatal care and associated factors among married men with wives who recently gave birth in Debre Tabor town, north West Ethiopia. BMC Pregnancy Childbirth. (2024) 24:642. doi: 10.1186/s12884-024-06809-0, PMID: 39363276 PMC11448043

[14] Sao SS Kisigo GA Osaki H Coleman JN Renju J Mwamba RN. Understanding male involvement in antenatal care in the Kilimanjaro region of Tanzania: barriers, facilitators, and opportunities for engagement. Sex Reprod Healthc. (2024) 39:100931. doi: 10.1016/j.srhc.2023.100931, PMID: 38039661 PMC11129671

[15] Xue WL He H-G Chua YJ Wang W Shorey S. Factors influencing first-time fathers' involvement in their wives' pregnancy and childbirth: a correlational study. Midwifery. (2018) 62:20–8. doi: 10.1016/j.midw.2018.03.002, PMID: 29627595

[16] Nakajima K Usui A Hayakawa Y. Feelings of older Japanese primiparous couples and satisfaction of older primiparous wives with their husbands’ support during pregnancy: focus on the perceptions of pregnant couples. Nurs Open. (2020) 7:1379–87. doi: 10.1002/nop2.509, PMID: 32802358 PMC7424439

[17] Kashanian M Faghankhani M YousefzadehRoshan M EhsaniPour M Sheikhansari N. Woman’s perceived stress during pregnancy; stressors and pregnancy adverse outcomes. J Matern Fetal Neonatal Med. (2021) 34:207–15. doi: 10.1080/14767058.2019.1602600, PMID: 30931659

[18] Kashanian M Faghankhani M Hadizadeh H Salehi MM Roshan MY Pour ME. Psychosocial and biological paternal role in pregnancy outcomes. J Matern Fetal Neonatal Med. (2020) 33:243–52. doi: 10.1080/14767058.2018.1488167, PMID: 29886805

[19] Charan J Kaur R Bhardwaj P Singh K Ambwani SR Misra S. Sample size calculation in medical research: a primer. Ann Natl Acad Med Sci. (2021) 57:74–080. doi: 10.1055/s-0040-1722104, PMID: 41022108

[20] Gibore NS Bali TA Kibusi SM. Factors influencing men’s involvement in antenatal care services: a cross-sectional study in a low resource setting, Central Tanzania. Reprod Health. (2019) 16:1–10. doi: 10.1186/s12978-019-0721-x 31072322 PMC6509760

[21] Odeny B McGrath CJ Langat A Pintye J Singa B Kinuthia J. Male partner antenatal clinic attendance is associated with increased uptake of maternal health services and infant BCG immunization: a national survey in Kenya. BMC Pregnancy Childbirth. (2019) 19:1–9. doi: 10.1186/s12884-019-2438-9 31395024 PMC6688227

[22] Rahman AE Perkins J Islam S Siddique AB Moinuddin M Anwar MR. Knowledge and involvement of husbands in maternal and newborn health in rural Bangladesh. BMC Pregnancy Childbirth. (2018) 18:1–12. doi: 10.1186/s12884-018-1882-2, PMID: 29914410 PMC6007056

[23] Chan KL Emery CR Fulu E Tolman RM Ip P. Association among father involvement, partner violence, and paternal health: UN multi-country cross-sectional study on men and violence. Am J Prev Med. (2017) 52:671–9. doi: 10.1016/j.amepre.2016.12.017 28209281

[24] Paul PL Pandey S. An examination of the factors associated with male partner attendance in antenatal care in India. BMC Pregnancy Childbirth. (2023) 23:532. doi: 10.1186/s12884-023-05851-8, PMID: 37481558 PMC10362642

[25] Heugh K Peter N Jackline A Ramecca M. Facilitators and barriers of male partner involvement in antenatal care at Kawempe National Referral Hospital. Int J Gynecol Obstet. (2025) [Epub ahead of Print]. doi: 10.1002/ijgo.70463, PMID: 40787825

[26] Abdiwali SA Adesina OA Fekadu GA Geta TG. Barriers and facilitators to antenatal care services utilisation in Somaliland: a qualitative study. BMJ Open. (2024) 14:e085073. doi: 10.1136/bmjopen-2024-085073, PMID: 39488416 PMC11535687

[27] Alio AP Bond MJ Padilla YC Heidelbaugh JJ Lu M Parker WJ. Addressing policy barriers to paternal involvement during pregnancy. Matern Child Health J. (2011) 15:425–30. doi: 10.1007/s10995-011-0781-1 21472512

[28] Firouzan V Noroozi M Farajzadegan Z Mirghafourvand M. Barriers to men's participation in perinatal care: a qualitative study in Iran. BMC Pregnancy Childbirth. (2019) 19:1–9. doi: 10.1186/s12884-019-2201-2 30691402 PMC6350307

[29] Mapunda B August F Mwakawanga D Mhando I Mgaya A. Prevalence and barriers to male involvement in antenatal care in Dar Es Salaam, Tanzania: a facility-based mixed-methods study. PLoS One. (2022) 17:e0273316. doi: 10.1371/journal.pone.0273316, PMID: 35984819 PMC9390926

[30] Morgan AK Awafo BA Quartey T Cobbold J. Husbands’ involvement in antenatal-related care in the Bosomtwe District of Ghana: inquiry into the facilitators and barriers. Reprod Health. (2022) 19:1–14. doi: 10.1186/s12978-022-01506-7 36456980 PMC9714231

[31] Chiang RKQ Shorey S. Men's experiences of antenatal care services in low-income and middle-income countries: a qualitative systematic review. Birth. (2023) 50:276–86. doi: 10.1111/birt.12688, PMID: 36309934

[32] Adejoh SO Olorunlana A Olaosebikan O. Maternal health: a qualitative study of male partners’ participation in Lagos, Nigeria. Int J Behav Med. (2018) 25:112–22. doi: 10.1007/s12529-017-9659-y, PMID: 28585072

[33] Ditekemena J Koole O Engmann C Matendo R Tshefu A Ryder R. Determinants of male involvement in maternal and child health services in sub-Saharan Africa: a review. Reprod Health. (2012) 9:32. doi: 10.1186/1742-4755-9-32, PMID: 23171709 PMC3573948

[34] Ali PA McGarry J Maqsood A. Spousal role expectations and marital conflict: perspectives of men and women. J Interpers Violence. (2022) 37:NP7082–108. doi: 10.1177/0886260520966667 33103547 PMC9092914

[35] Kaye DK Kakaire O Nakimuli A Osinde MO Mbalinda SN Kakande N. Male involvement during pregnancy and childbirth: men’s perceptions, practices and experiences during the care for women who developed childbirth complications in Mulago hospital, Uganda. BMC Pregnancy Childbirth. (2014) 14:54. doi: 10.1186/1471-2393-14-54, PMID: 24479421 PMC3916059

[36] Zakaria M Khan AZR Ahmad MS Cheng F Xu J. Women’s perception of male involvement in antenatal, childbirth and postnatal care in urban slum areas in Bangladesh: a community-based cross-sectional study. Healthcare. (2021) 9:473. doi: 10.3390/healthcare9040473 33923575 PMC8073583

[37] Maluka SO Peneza AK. Perceptions on male involvement in pregnancy and childbirth in Masasi District, Tanzania: a qualitative study. Reprod Health. (2018) 15:68. doi: 10.1186/s12978-018-0512-9, PMID: 29678184 PMC5910565

[38] Rumaseuw R Berliana S Nursalam N Efendi F Pradanie R Rachmawati P. Factors affecting husband participation in antenatal care attendance and delivery. IOP Conf Ser Earth Environ Sci. (2018) 10:1755–315. doi: 10.1088/1755-1315/116/1/012012

[39] Lowe M. Social and cultural barriers to husbands’ involvement in maternal health in rural Gambia. Pan Afr Med J. (2017) 27:1–7. doi: 10.11604/pamj.2017.27.255.11378, PMID: 29187924 PMC5660305

[40] Sodeinde KJ Amoran OE Abiodun OA. Male involvement in birth preparedness in Ogun state, Nigeria: a rural/urban comparative cross-sectional study. Afr J Reprod Health. (2020) 24:70–84. doi: 10.29063/ajrh2020/v24i2.7 34077093

[41] Cameron RP Wells JD Hobfoll SE. Stress, social support and coping in pregnancy: taking gender and ethnicity into account. J Health Psychol. (1996) 1:195–208. doi: 10.1177/135910539600100204, PMID: 22011704

[42] Kakaire O Kaye DK Osinde MO. Male involvement in birth preparedness and complication readiness for emergency obstetric referrals in rural Uganda. Reprod Health. (2011) 8:1–7. doi: 10.1186/1742-4755-8-12 21548976 PMC3118172

