# Supplementary material for: Perceptions and satisfaction among couples on male involvement during pregnancy: a cross-sectional study
Source: Front Public Health. 2025 Oct 22;13:1625849. doi: 10.3389/fpubh.2025.1625849 (PMC12586082; doi:10.3389/fpubh.2025.1625849)
Supplement: Supplementary file 1 [file Supplementary_file_1.docx]

**Questionnaire on Male Involvement During Pregnancy**

**Instructions:**

- Men: Please answer based on your own involvement.
- Women: Please answer based on your husband’s involvement.
- For each item:
  - Participation: Yes / No
  - Satisfaction: very satisfied = 5 points, satisfied = 4 points, average = 3 points, dissatisfied = 2 points, and very dissatisfied = 1 point

**1.** **Have you/your husband accompanied your wife/husband to antenatal care visits?**

- Yes
- No

**1.1 Satisfaction:**

- 5
- 4
- 3
- 2
- 1

**2. Have you/your husband discussed the results of antenatal care with your wife/husband?**

- Yes
- No

**2.1 Satisfaction:**

- 5
- 4
- 3
- 2
- 1

**3. Have you/your husband consulted medical personnel regarding antenatal care?**

- Yes
- No

**3.1 Satisfaction:**

- 5
- 4
- 3
- 2
- 1

**4. Have you/your husband provided financial support to your wife/husband during pregnancy?**

- Yes
- No

**4.1 Satisfaction:**

- 5
- 4
- 3
- 2
- 1

**5. Have you/your husband provided transportation support to your wife/husband during pregnancy?**

- Yes
- No

**5.1 Satisfaction:**

- 5
- 4
- 3
- 2
- 1

**6. Have you/your husband provided nutritional support to your wife/husband during pregnancy?**

- Yes
- No

**6.1 Satisfaction:**

- 5
- 4
- 3
- 2
- 1

**7. Have you/your husband provided emotional support to your wife/husband during pregnancy?**

- Yes
- No

**7.1 Satisfaction:**

- 5
- 4
- 3
- 2
- 1

**8. Have you/your husband provided household support to your wife/husband during pregnancy?**

- Yes
- No

**8.1 Satisfaction:**

- 5
- 4
- 3
- 2
- 1

**9. Have you/your husband participated in decision-making regarding the mode of birth?**

- Yes
- No

**9.1 Satisfaction:**

- 5
- 4
- 3
- 2
- 1

**10. Have you/your husband attended antenatal health education sessions together?**

- Yes
- No

**10.1 Satisfaction:**

- 5
- 4
- 3
- 2
- 1

**11. Have you/your husband actively learned about pregnancy-related knowledge?**

- Yes
- No

**11.1 Satisfaction:**

- 5
- 4
- 3
- 2
- 1
